# Applying a novel two-step deep learning network to improve the automatic delineation of esophagus in non-small cell lung cancer radiotherapy

**DOI:** 10.3389/fonc.2023.1174530

**Published:** 2023-07-18

**Authors:** Fuli Zhang, Qiusheng Wang, Na Lu, Diandian Chen, Huayong Jiang, Anning Yang, Yanjun Yu, Yadi Wang

**Affiliations:** ^1^ Radiation Oncology Department, The Seventh Medical Center of Chinese People's Liberation Army (PLA) General Hospital, Beijing, China; ^2^ School of Automation Science and Electrical Engineering, Beihang University, Beijing, China

**Keywords:** organs at risk, medical image segmentation, DenseNet, spatial and channel cascaded attention, non-small cell lung cancer

## Abstract

**Purpose:**

To introduce a model for automatic segmentation of thoracic organs at risk (OARs), especially the esophagus, in non-small cell lung cancer radiotherapy, using a novel two-step deep learning network.

**Materials and methods:**

A total of 59 lung cancer patients’ CT images were enrolled, of which 39 patients were randomly selected as the training set, 8 patients as the validation set, and 12 patients as the testing set. The automatic segmentations of the six OARs including the esophagus were carried out. In addition, two sets of treatment plans were made on the basis of the manually delineated tumor and OARs (Plan1) as well as the manually delineated tumor and the automatically delineated OARs (Plan2). The Dice similarity coefficient (DSC), 95% Hausdorff distance (HD95), and average surface distance (ASD) of the proposed model were compared with those of U-Net as a benchmark. Next, two groups of plans were also compared according to the dose–volume histogram parameters.

**Results:**

The DSC, HD95, and ASD of the proposed model were better than those of U-Net, while the two groups of plans were almost the same. The highest mean DSC of the proposed method was 0.94 for the left lung, and the lowest HD95 and ASD were 3.78 and 1.16 mm for the trachea, respectively. Moreover, the DSC reached 0.73 for the esophagus.

**Conclusions:**

The two-step segmentation method can accurately segment the OARs of lung cancer. The mean DSC of the esophagus realized preliminary clinical significance (>0.70). Choosing different deep learning networks based on different characteristics of organs offers a new option for automatic segmentation in radiotherapy.

## Introduction

According to global cancer statistics in 2020, lung cancer is the number one malignant tumor in China in terms of incidence and the number one cause of cancer deaths (1). Non-small cell lung cancer (NSCLC) occupies the majority of lung cancer. Radiation therapy (RT) plays an important role in the whole process of lung cancer treatment (2, 3). In a radiotherapy routine clinical workflow, a doctor manually contours the tumor and organs at risk (OARs) according to the information from multimodal medical images like CT and MRI. MRI can clearly display lesions, lymph nodes, and pleural lesions in the mediastinum, providing important information for target delineation; CT is commonly used for OAR segmentation in clinic. This process usually requires a lot of time and energy of doctors, and the segmentation quality depends on the prior knowledge and experience of doctors to a large extent. Inter- and intraobserver segmentation inconsistencies in tumor and OARs have been reported (4–9). Therefore, increasing the efficiency and consistency of contour segmentation has become imperative.

Today, automatic tumor and OAR segmentation based on deep learning has become one of the hotspots in radiotherapy. Ronneberger O et al. (10) proposed a convolutional neural network (CNN) named U-Net, which has a symmetric architecture for medical image segmentation. The encoding–decoding symmetric architecture of U-Net has become the classic framework for image segmentation. Zhang GB et al. (11) developed a dual path network model nnU-Net for both OAR and tumor segmentation based on the basic structure of Unet. Ashok M et al. (12) integrated the InceptionV3 module in Unet to construct U-Net InceptionV3, which segments the esophagus, heart, trachea, and aorta. Zhang J et al. (13) proposed the multi-output fully convolutional network (MOFCN) network, which designed a backbone network and three branch network structures based on the different characteristics of the lungs, heart, and spinal cord. He KM et al. proposed a residual network named ResNet (14). By transforming the learning object of some layers into learning residual functions, this mapping highlights the tiny input changes and alleviates the gradient disappearance problem caused by the increase in depth. Next, Huang G et al. proposed the DenseNet network (15). Several dense blocks are linked with a transition layer in DenseNet, and the channels of each dense block feature map are concatenated in series to increase the number of feature maps and improve the utilization of feature maps. Cao Z et al. (16) proposed a dense-connected SE ResUnet based on a coarse and fine two-step segmentation method.

In our last study, a model established on modified DenseNet network was proposed (17), and the bilateral lung, spinal cord, heart, and trachea were accurately contoured except for the esophagus. The small size of the esophagus, low contrast to neighboring tissues, individualized differences in air filling, and certain mobility make it difficult to automatically delineate (18–21). In this study, a novel two-step deep learning model is proposed to focus on automatically delineating the esophagus in two-dimensional (2D) CT images. The performance of the proposed model was compared with that of U-Net in terms of geometric metrics of DSC, HD95, and ASD. Then, dosimetric metrics including hetereogeneity index (HI), conformity index (CI) of the target, Dmax, Dean and Vx of manually and automatically delineated OAR were compared.

## Materials and methods

### Data acquisition and preprocessing

A total of 59 lung cancer patients’ CT images at the Seventh Medical Center of Chinese PLA General Hospital were collected. All patients received contrast agent during CT acquisition. The CT images were acquired on a CT simulator (Brilliance Big Bore, Philips Medical Systems, Madison, WI) from the larynx level to the bottom of the lungs with a 5-mm slice thickness in the helical scan mode. The study was approved by the ethics committee of the Seventh Medical Center of Chinese PLA General Hospital. All patients provided written consent for the storage of their medical information in the hospital database. The gray value of the original CT images with a resolution of 512 × 512 was mapped to the range of 0–255. The window width and level were set to 400 and 40, respectively. The OARs were delineated by an experienced radiation oncologist who specializes in the thoracic region and were then peer-reviewed by two other experts. These manual delineations were used to generate the ground truth (GT) in this study. The OARs were filled in different gray values to create mask images as the training labels.

The training dataset was composed of 2,335 CT images of 39 patients. The validation dataset comprised 617 images of 8 patients. The testing dataset included 854 images of 12 patients. Detailed information is shown in **Table 1**. The datasets are mainly composed of stage III and IV patients who are more serious. These CT images were sent to the network after data cleaning and augmentation.

**Table 1:** Dataset information.

Characteristics	Training set	Validation set	Testing set
No. of patients	39	8	12
Tumor site, right lung:left lung	19:20	5:3	8:4
Stage at diagnosis	I = 0; II = 5; III = 9; IV = 25	I = 0; II = 0; III = 2; IV = 6	I = 0; II = 2; III = 5; IV = 5
Lobe location			
Upper left	18	3	4
Lower left	2	0	0
Upper right	8	3	3
Middle right	4	1	1
Lower right	7	1	4
Pathological type			
Squamous cell carcinoma: adenocarcinoma	23:16	4:4	7:5

The deep learning framework of this study is TensorFlow-graphics processing unit (GPU) 1.7.0, the GPU is GTX 1070 Ti, and the video memory is 8 GB.

### Two-step automatic segmentation method

Different OARs have different characteristics; thus, the segmentation difficulty is different. For example, the volumes of the heart, left lung, and right lung are large; the anatomical positions of the trachea and spinal cord are relatively fixed; and the boundary with neighboring structures is clear; thus, the segmentation of these OARs is easier. However, the filling degree of the esophagus varies with each individual, and the edge is fuzzy as well; hence, the esophagus is more difficult to segment. Therefore, different networks need to be chosen to handle different segmentation tasks.

**Figure 1:**
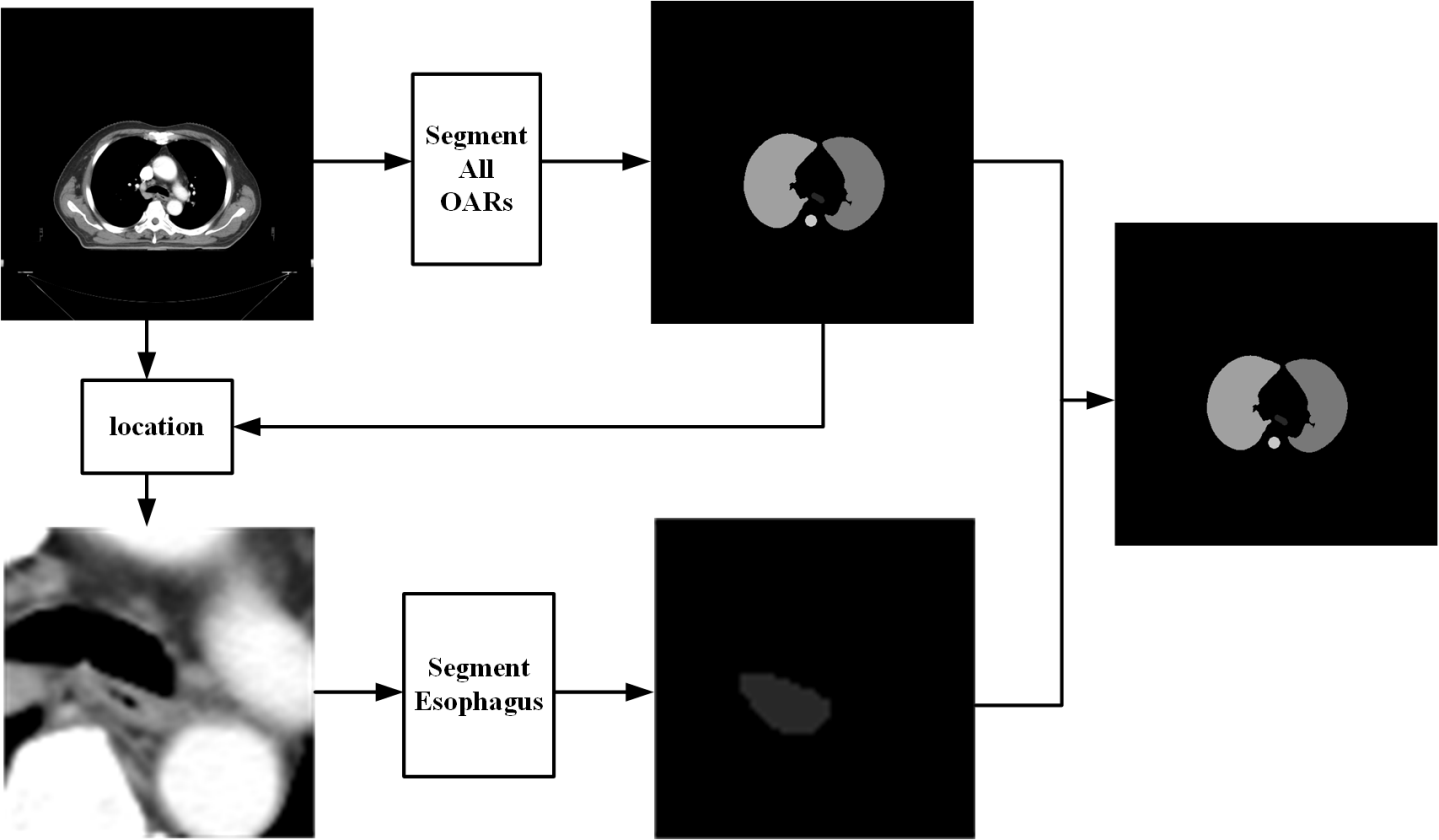
Workflow diagram of the proposed method.

Here, a two-step method for segmenting OARs of NSCLC is proposed. In the first step, DenseNet is fed full-size CT images to segment five OARs: the left lung, right lung, heart, spinal cord, and trachea. According to the position of the trachea in this step, regions of the CT images, which include the trachea and esophagus, are located. The second step uses the residual attention network to increase the segmentation accuracy of the esophagus. Finally, the output is corrected according to the results in the second step. The workflow diagram is shown in **Figure 1**.

### DenseNet model for the first step segmentation

DenseNet67 is trained to achieve accurate automatic segmentation of five OARs, including the left lung, right lung, heart, spinal cord, and trachea. The specific architecture and training process of the network can be found in our published article (17).

### Residual attention network model for the second step segmentation

The main purpose of this study is to improve the accuracy of automatic esophageal delineation; thus, a residual attention network was proposed in the second step segmentation. Considering that the esophagus is usually adjacent to the trachea, the average center of gravity of the trachea in the CT image is calculated. Then, with this center of gravity as the center, the corresponding area was intercepted and sent to the residual attention network. The residual attention network uses the residual blocks of spatial and channel cascaded attention, which has a good effect on targets with small volumes and fuzzy boundaries.

**Figure 2:**
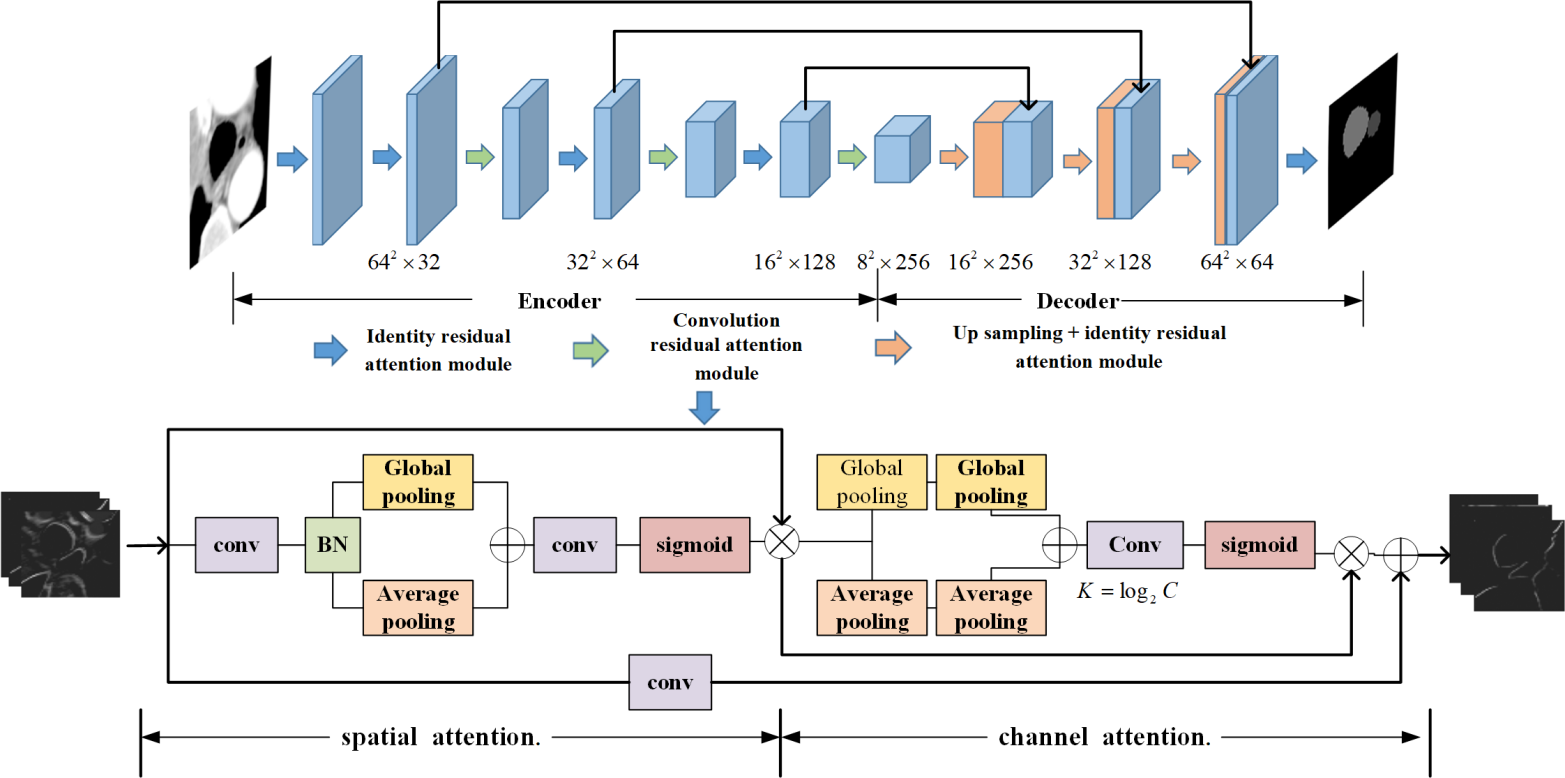
Residual attention network and attention module diagram.

The specific architecture of the residual attention network and attention module is shown in **Figure 2**. The overall structure is similar to DenseNet67 and is also divided into two parts: encoder and decoder. The convolution residual attention module and identity residual attention module are used alternately in the encoding stage, and the upsampling layers and identity residual attention module are used alternately in the decoding stage.

The residual attention network introduces a residual block of spatial and channel cascaded attention. This residual attention block simulates the process of manually segmenting the object and allocates more attention to the region where the esophagus may be located in the image. The whole network is composed of multiple attention residual blocks. The output of the identity residual attention module is directly connected to the input rather than through the convolution layer. The overall residual attention network structure is similar to DenseNet, and the U-shaped structure is selected. Each layer of the coding path is connected with the parsing path through a long connection.

The attention layer in the attention residual block is composed of two parts: spatial attention and channel attention. In the spatial attention stage, when the feature map with the C × W × H dimension enters the module, each channel of the feature map is first pooled to the global maximum, and then each channel of the feature map is pooled to the global average and compressed into two 1 × W × H feature maps. The significant information and average information are measured at the spatial scale, and then, the two feature maps are aggregated and sent into the convolution layer. The probability value of the possible area of each pixel is obtained through the activation function and finally multiplied back to the feature map so that the area of the esophagus is easier to activate by the next layer. Attention to the channel of the feature map means that the feature map of C × W × H through the first step of spatial attention carries out global maximum pooling of the channel dimension along with the width and height directions. The global average pooling of the channel dimension is compressed into two C × 1 × 1 columns to obtain the significant information and average information at the channel scale. The number of channels C will increase with the deepening of the number of layers; a variable scale K × 1 × 1 convolution kernel is used, where *K* = log_2_ *C*. With the increase in the number of channels in the feature map, the receptive field also increases to realize the information fusion between channels, obtain the probability value of each channel containing region of interest information, multiply the probability of C × 1 × 1 back to the C × W × H feature map, and give more weight to the channels with significant features.

### Training data for the residual attention network

The second step takes the center of gravity of the trachea in the label as the center, intercepting 64 × 64 regions from the above 39 training datasets. To be close to the segmentation scene in the second step, the position of the center of gravity is randomly offset in the range of 0–4 mm by referring to the average surface distance (ASD) between the esophagus segmented by the neural network and the label in the first step. Considering that the esophagus is usually below the trachea in the image, the position change in the esophagus from the beginning to the bronchial intersection is not obvious. From the bronchial intersection to the cardia, the esophagus is offset from the center to the left in the CT image. To ensure that the intercepted image region includes the esophagus, the center of gravity is shifted to the left by 5 mm and upward by 5 mm, leaving sufficient space for the esophagus. If the esophagus is not included in the CT image, it is offset by 1 mm layer by layer based on the center of gravity coordinates of the previous layer.

### Accuracy evaluation metrics

The DSC, HD95, and ASD are used to evaluate the automatic segmentation accuracy (22). The segmentation results of OARs obtained by using the two-step segmentation model were compared with those obtained by using the U-Net as the baseline.

To evaluate the dosimetric impact of the proposed automatic segmentation method, 12 pairs of 7-field intensity-modulated radiotherapy (IMRT) plans were designed for patients in the testing set using GT planning target volume (PTV) and OARs (Plan1) as well as GT PTV and automatically segmented OARs (Plan2). The dose–volume histogram parameter differences between manually delineated OARs and automatically segmented OARs are calculated. All 12 pairs of plans are prescribed to 2 Gy per fraction for 30 fractions and normalized as 100% prescription dose to 95% of PTV. The HI and CI of the PTV are calculated according to the formula in reference (23). The differences in HI, CI, Dmax, Dmean, and Vx between the two groups of plans are used to evaluate the clinical feasibility of the proposed model.

### Statistical analysis

SPSS statistical software (version 20.0, SPSS Inc., Chicago, IL, USA) was used for statistical analysis. Wilcoxon’s signed rank test and Students’ *t*-test are used to compare the differences in geometric and dosimetric metrics. Quantitative data are expressed as the mean ± standard deviation ( $\bar{x}\pm s$ ), and a value of P< 0.05 was considered statistically significant.

## Results

### Geometric metrics

In this study, after the first step of segmentation, the average DSC values of the bilateral lung, heart, spinal cord, and trachea were 0.92, 0.94, 0.89, 0.87, and 0.81, respectively, but the average DSC value of the esophagus was below 0.70. Therefore, the second step of segmentation was required to improve segmentation of the esophagus.

**Figure 3:**
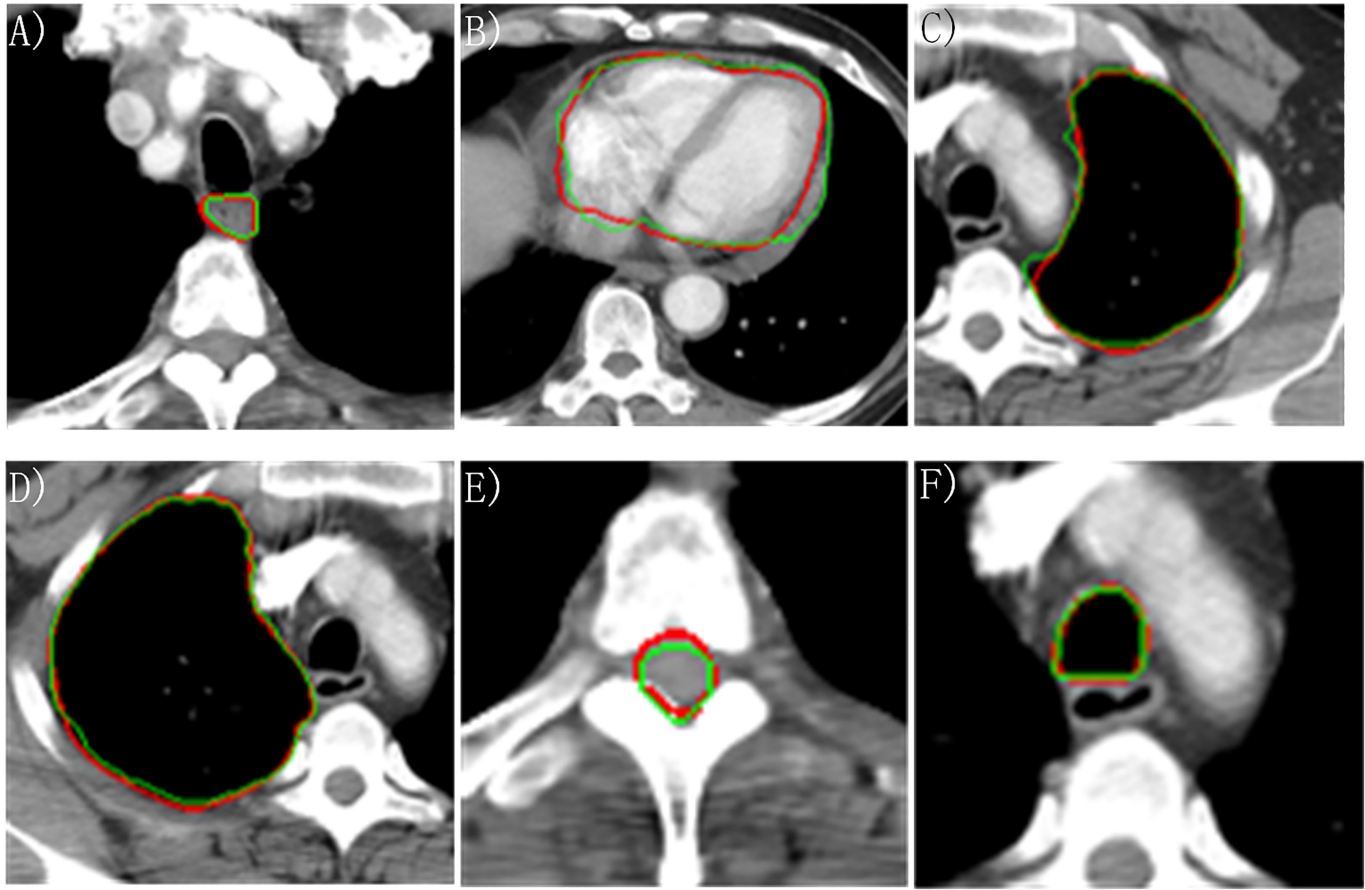
Manually and automatically segmented organs at risk based on the proposed model. Red line: manual contour; green line: automatic contour. **(A)** Esophagus; **(B)** Heart; **(C)** Left lung; **(D)** Right lung; **(E)** Cord; **(F)** Trachea.

**Figure 4:**
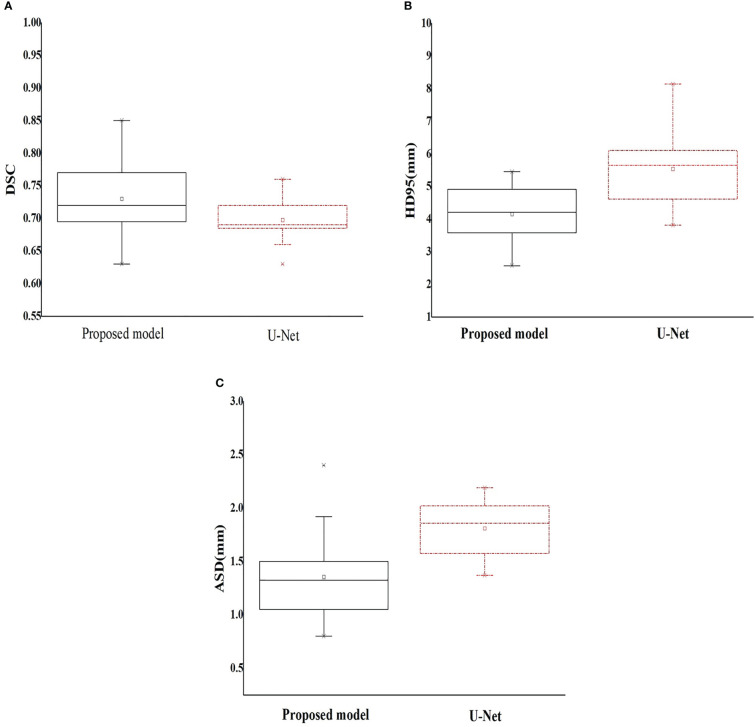
Box plot of the geometric metrics of the esophagus (**(A)** Dice similarity coefficient; **(B)** 95% Hausdorff distance; and **(C)** average surface distance).

The DSC, HD95, and ASD based on the proposed two-step segmentation model and U-Net are listed in **Table 2**. **Figure 3** demonstrates the comparison of the results between manual and automatic segmentation based on the proposed model for a typical patient. Moreover, **Figure 4** specifically shows the DSC, HD95, and ASD of the esophagus in the testing set.

**Table 2:** Comparison of geometric parameters of two methods ( $\bar{x}\pm s$ ).

DSC						
	Esophagus	Heart	Right lung	Left lung	Cord	Trachea
Proposed	0.73 ± 0.06	0.89 ± 0.03	0.92 ± 0.03	0.94 ± 0.02	0.87 ± 0.01	0.81 ± 0.03
U-Net	0.69 ± 0.03	0.85 ± 0.04	0.88 ± 0.04	0.90 ± 0.56	0.82 ± 0.04	0.81 ± 0.06
*P* value	0.114	0.017	0.023	0.026	0.000	0.906
HD95 (mm)						
Proposed	4.32 ± 1.02	8.89 ± 3.1	11.92 ± 4.56	10.77 ± 3.25	7.09 ± 0.38	3.78 ± 0.87
U-Net	5.53 ± 1.26	8.53 ± 1.6	11.10 ± 2.88	11.67 ± 2.74	8.64 ± 1.23	7.30 ± 1.81
*P* value	0.017	0.000	0.604	0.470	0.462	0.000
ASD (mm)						
Proposed	1.35 ± 0.45	2.77 ± 1.34	2.42 ± 0.69	2.05 ± 0.66	1.70 ± 0.42	1.16 ± 0.36
U-Net	1.81 ± 0.27	2.36 ± 0.40	2.70 ± 0.63	2.69 ± 0.71	2.10 ± 0.54	1.87 ± 0.47
*P* value	0.008	0.000	0.414	0.032	0.073	0.000

### Dosimetric metrics

The dose–volume parameters of the OARs based on manual and automatic segmentation are listed in **Table 3**. There were no statistically significant differences between the dosimetric parameters of manual and automatically delineated OARs (P > 0.05). The CIs of PTV in Plan1 and Plan2 were 0.67 ± 0.05 and 0.68 ± 0.04, respectively, while the HIs of PTV in Plan2 were 0.12 ± 0.06 and 0.11 ± 0.06, respectively. The differences in CI were not statistically significant (P > 0.05). Although the difference in HI was statistically significant (P< 0.05), it was very small.

**Table 3:** Comparison of dosimetric parameters of the planning target volume and organs at risk between manual and automatic segmentation–based plans ( $\bar{x}\pm s$ ).

Dosimetric parameters		Plan1	Plan2	*P* value
PTV	CI	0.67 ± 0.05	0.68 ± 0.04	0.071
	HI	0.12 ± 0.06	0.11 ± 0.06	0.005
Spinal cord	Dmax (Gy)	37.40 ± 12.32	36.92 ± 12.34	0.773
Heart	V30 (%)	19.42 ± 15.49	18.5 ± 16.08	0.119
	V40 (%)	13.17 ± 11.75	12.25 ± 11.57	0.152
	Dmean (Gy)	15.13 ± 9.94	14.32 ± 10.30	0.073
Lung all	V5 (%)	51.42 ± 20.59	50.5 ± 20.77	0.794
	V10 (%)	40.83 ± 17.35	40.08 ± 17.43	0.169
	V20 (%)	23.75 ± 11.51	23.33 ± 11.28	0.269
	V30 (%)	14.08 ± 7.24	13.75 ± 7.28	0.394
	Mean (Gy)	13.09 ± 5.19	12.83 ± 5.24	0.088
Trachea	Dmean	28.30 ± 20.74	28.03 ± 20.98	0.713
Esophagus	Dmean	27.12 ± 13.75	25.96 ± 13.29	0.127

## Discussion

According to the benchmark study of Yang JZ, et al. (22), the OAR with the highest DSC is the lung, with an average value between 0.95 and 0.98, while the organ with the lowest DSC is the esophagus, with a range of 0.55–0.72. The results in this study are relatively consistent with those of the above study with the lung having the highest DSC. In particular, the esophagus in our study achieved a better average DSC (range: 0.63–0.85).

Lustberg T et al. (24) used a prototype of deep learning automatic segmentation software (Mirada) to generate thoracic OARs. This prototype uses a deep learning model based on a 2D multiclass CNN, and 450 lung patients were used to train the model. The median DSCs of the spinal cord, lungs, and heart were 0.83, >0.95, and >0.90, respectively. Zhang T et al. (25) developed a 2D automatic segmentation CNN (AS-CNN) based on the ResNet101 network using a dataset of 250 lung cancer patients and achieved the average DSCs of 0.94, 0.89, 0.94, 0.82, and 0.73 for the left lung, heart, right lung, spinal cord, and esophagus. In particular, the training datasets used less cases in this study (59 vs. 250), DenseNet has a strong ability of feature extraction for small samples, and the segmentation results are similar to those of the training model using larger datasets. He T et al. (26) developed a uniform U-like encoder–decoder architecture based on U-Net and trained it for two task learning schema. High DSC values were obtained for the esophagus (0.86), heart (0.95), trachea (0.92), and aorta (0.95). Vesal S et al. (27) generated a deep learning framework to segment the heart, esophagus, trachea, and aorta. Dilated convolutions and aggregated residual connections in the bottleneck of a 2D U-Net-styled network were used to incorporate global context and dense information and scored the mean DSCs of 0.94, 0.86, 0.93, and 0.94 for the heart, esophagus, trachea, and aorta. Han M et al. (28) developed a novel framework called multiresolution VB-Net based on the V-Net architecture to segment the esophagus, heart, trachea, and aorta and obtained DSCs of 0.87, 0.95, 0.93, and 0.95, respectively.

The U-Net-generative adversarial network (U-Net-GAN) proposed by Dong X et al. (29) trained 35 cases and segmented five thoracic OARs. Of them, the left lung, right lung, and heart were autosegmented by a 2.5D GAN model, while the esophagus and spinal cord were autosegmented by a three-dimensional (3D) GAN model. The DSCs of the left and right lungs, spinal cord, esophagus, and heart were 0.97,0.97, 0.90, 0.75, and 0.87, respectively. The ASD was in the range of 0.4 and 1.5 mm, and the HD95 was between 1.2 and 4.6 mm. Zhu JH et al. (30) developed an automatic segmentation model to segment OARs of lung cancer cases. In their study, a U-shaped network with a 3D convolution kernel was used, the HD95 lied in the range of 7.96 and 8.74 mm, and the ASD lied in the range of 1.81 and 2.92 mm. Based on 3D U-Net, Feng X et al. (31) proposed a new model to segment thoracic OARs. In their study, given that each organ has a relatively fixed position within the CT images, the original 3D images were first cropped into smaller patches to ensure that each patch contained only one organ to be segmented. Then, for each organ, an individual 3D U-Net was trained to segment the organ from the cropped patches. The DSCs reached 0.89, 0.97, 0.98, 0.93, and 0.73, respectively, for the spinal cord, right lung, left lung, heart, and esophagus. Van Harten L et al. (32) obtained the best DSC and HD among all the methods based on CNN architecture by combining a 2D CNN with a 3D CNN. The DSCs of 0.84, 0.94, 0.91, and 0.93 and HD of 3.4, 2.0, 2.1, and 2.7 mm were achieved for the esophagus, heart, trachea, and aorta, respectively.

To date, three main development directions exist in medical image segmentation. The first is to deepen the network depth, extract deeper semantic features to obtain stronger network expression ability, or widen the network to increase the number of channels to obtain more details in the same layer, such as texture features of different frequencies and boundary features in different directions. The second is to achieve a more effective spatial feature extraction ability by learning the sequence association properties of multiple CT levels of a patient, represented by 3D U-Net and many other derivative networks. The third represented by DenseNet is to improve the utilization of the feature map by sharing the layer-by-layer feature map, so as to enhance the feature expression ability and improve the generalization of the network (33).

In this study, the segmentation results of the left lung and right lung were better than those of the spinal cord, heart, esophagus, and trachea. Observing the CT image, we can see that there are clear boundaries between the left lung and the right lung in the original image. Compared with the bilateral lung, although the spinal cord has a bone structure as support and texture and edges are demarcated, it accounts for less area in the image. The number of negative samples in the image background is much larger than the number of positive samples in the spinal cord. The imbalance of positive and negative samples leads to the relatively low accuracy of the spinal cord.

The heart is located in the center of the slice, and there are other organs around it, such as the lung and esophagus. The image features of the center are not strong; thus, the segmentation result is slightly worse than that of the lung.

The features of the trachea are similar to those of the lung, which has a relatively certain position and clear boundary with other tissues except the esophagus. A deep learning network is more inclined to extract significant information in gradient propagation and has the tendency to misjudge the esophagus as trachea that is very close; thus, the segmentation result of the trachea is not as good as that of the lung.

The filling degree of the esophagus is different, the image area is small, there is no bone structure to support it, and there are tracheas with similar size or shape next to the esophagus. A deep learning network cannot extract similar features effectively using small sample training; hence, the segmentation effect of the esophagus needs to be further improved by increasing the sample size in the next step, although the automatic delineation of esophagus has preliminary clinical significance in this study.

## Conclusion

Compared with U-Net, the two-step segmentation model has a more stable automatic segmentation effect and better generalization performance. The average DSC of the proposed model is higher, and the variance is small. HD95 is a metric to measure the maximum distortion of segmentation results, and its size is affected by the number of outliers. The HD95 and ASD of the proposed model are better than those of U-Net, which demonstrates that the two-step segmentation method has better continuity and produces fewer outliers.

Even if the training set has fewer images, it can still effectively prevent the occurrence of overfitting because the residual attention network has a strong feature extraction ability in the training of small samples. Additionally, it can effectively alleviate the problem of gradient disappearance in the training process by repeatedly using different levels of feature maps. It provides a new idea for medical image segmentation.

## Data availability statement

The raw data supporting the conclusions of this article will be made available by the authors, without undue reservation.

## Ethics statement

Written informed consent was obtained from the individual(s) for the publication of any potentially identifiable images or data included in this article.

## Author contributions

FZ and QW contributed conception and design of the study. FZ and AY trained the deep learning models, FZ and QW performed data analysis and drafted the manuscript. NL, DC, HJ, and YW helped to collect the data and evaluate radiotherapy planning. YY designed radiotherapy planning. All authors contributed to the article and approved the submitted version.
